# Prescribing of Statins After Lower Extremity Revascularization Procedures in the US

**DOI:** 10.1001/jamanetworkopen.2021.36014

**Published:** 2021-12-03

**Authors:** Nikhil Singh, Li Ding, Justin Devera, Gregory A. Magee, Parveen K. Garg

**Affiliations:** 1 Section of Cardiology, Department of Medicine, The University of Chicago, Chicago, Illinois; 2 Department of Preventive Medicine, University of Southern California Keck School of Medicine, Los Angeles; 3 Department of Internal Medicine, University of Southern California Keck School of Medicine, Los Angeles; 4 Division of Vascular Surgery and Endovascular Therapy, University of Southern California Keck School of Medicine, Los Angeles; 5 Division of Cardiology, University of Southern California Keck School of Medicine, Los Angeles

## Abstract

**Importance:**

The use of statins in patients with symptomatic peripheral artery disease remains suboptimal despite strong clinical practice guideline recommendations; however, it is unknown whether rates are associated with substantial improvements after lower extremity revascularization.

**Objective:**

To report longitudinal trends of statin use in patients with peripheral artery disease undergoing lower extremity revascularization and to identify the clinical and procedural characteristics associated with prescriptions for new statin therapy at discharge.

**Design, Setting, and Participants:**

This was a retrospective cross-sectional study using data from the Vascular Quality Initiative registry of patients who underwent lower extremity peripheral artery disease revascularization from January 1, 2014, through December 31, 2019. The Vascular Quality Initiative is a multicenter registry database including academic and community-based hospitals throughout the US. Patients aged 18 years or older undergoing lower extremity revascularization with available statin data (preprocedure and postprocedure) were included. Those not receiving statin therapy for medical reasons were excluded from final analyses.

**Exposures:**

Patients undergoing lower extremity revascularization for whom statin therapy is indicated.

**Main Outcomes and Measures:**

Multivariate logistic regression was used to determine the clinical and procedural characteristics associated with new statin prescription for patients not already taking a statin preprocedure. The overall rates of statin prescription as well as rates of new statin prescription at discharge were determined. In addition, the clinical, demographic, and procedural characteristics associated with new statin prescription were analyzed.

**Results:**

There were 172 025 procedures corresponding to 125 791 patients (mean [SD] age, 67.7 [11.0] years; 107 800 men [62.7%]; and 135 405 White [78.7%]) included in the analysis. Overall rates of statin prescription at discharge improved from 17 299 of 23 093 (75%) in 2014 to 29 804 of 34 231 (87%) in 2019. However, only 12 790 of 42 020 patients (30%) not already taking a statin at the time of revascularization during the study period were newly discharged with a statin medication. New statin prescription rates were substantially lower after endovascular intervention (7745 of 29 581 [26%]) than after lower extremity bypass (5045 of 12 439 [41%]). Body mass index of 30 or greater (odds ratio [OR], 1.13; 95% CI, 1.04-1.24; *P* < .001), diabetes (diet-controlled vs no diabetes, OR, 1.22; 95% CI, 1.05-1.41; *P* = .01), smoking (current vs never, OR, 1.32; 95% CI, 1.21-1.45; *P* < .001), hypertension (OR, 1.19; 95% CI, 1.09-1.29; *P* < .001), and coronary heart disease (OR, 1.26; 95% CI, 1.17-1.35; *P* < .001) were associated with an increased likelihood of new statin prescription after endovascular intervention, whereas female sex, older age, antiplatelet use, and prior peripheral revascularization were associated with a decreased likelihood.

**Conclusions and Relevance:**

In this cross-sectional study, although statin use was associated with a substantial improvement after lower extremity revascularization, more than two-thirds of patients not already taking a statin preprocedure remained not taking a statin at discharge. Further investigations to understand the clinical implications of these findings and develop clinician- and system-based interventions are needed.

## Key Points

**Question:**

In patients undergoing lower extremity revascularization, what are the rates of new statin prescription after the procedure and the clinical characteristics associated with statin prescription?

**Findings:**

In this cross-sectional study of 125 791 patients presenting for peripheral revascularization within the Vascular Quality Initiative registry, only 30% of patients not receiving statin therapy at the time of intervention were discharged with a new statin prescription. Traditional cardiac risk factors and known cardiac disease were associated with new statin prescription.

**Meaning:**

This study suggests that statin prescription remains suboptimal and may be associated with other comorbid conditions despite evidence of benefit in all individuals with revascularized peripheral artery disease.

## Teaser

This cross-sectional study uses data from the Vascular Quality Initiative to assess the rates of new statin prescription and the associated clinical characteristics of patients after lower extremity revascularization.

## Introduction

The use of antiplatelet and statin therapy in individuals with lower extremity (LE) peripheral artery disease (PAD) for the prevention of cardiovascular morbidity and mortality is well established and endorsed in current clinical practice guidelines.^1,2^ Statin therapy is associated with a reduction in major vascular events, symptom progression, and the need for peripheral revascularization.^3,4,5^ Statin therapy reduces all-cause mortality and adverse limb events in patients with LE PAD.^6^

Despite these benefits and the recommendation for their use, statin therapy in patients with PAD remains suboptimal, even in those whose PAD has progressed to the point of requiring revascularization.^7^ The presence of coronary disease or comorbid cardiac conditions appears most related to the likelihood of statin use before revascularization.^8^ A previous study^9^ in a Veterans Affairs cohort reported that 79% of patients with PAD were prescribed any statin therapy, and only 41% of statins were prescribed at guideline-appropriate intensity. For patients with isolated PAD, these numbers decrease to 69% and 29%, respectively. Other studies have reported even lower rates in those with isolated PAD.^10,11^

Data regarding rates of statin use after revascularization in patients with PAD are limited.^12^ Presentation for LE revascularization represents a potentially high-yield opportunity to address statin underuse in individuals who are symptomatic for PAD and whose condition may have been previously unidentified by their clinician. Identification of these individuals is particularly important given the finding that initiation of statin therapy after revascularization results in a significant reduction of all-cause mortality, amputation, and symptoms.^13^ Using the Society of Vascular Surgery (SVS) Vascular Quality Initiative (VQI), we aimed to determine longitudinal trends in discharge statin prescription rates for patients undergoing LE revascularization. We also aimed to evaluate the clinical, demographic, and procedural characteristics associated with de novo statin prescription.

## Methods

### Study Participants

We performed a retrospective cross-sectional study using the peripheral vascular intervention, suprainguinal LE bypass (LEB), and infrainguinal LEB databases of the VQI registry. The VQI is a large, multicenter national registry database in the US created by the SVS with support from the American College of Cardiology; the VQI has the aim of improving vascular health care through the collection and exchange of information among regional quality groups.^14^ Physicians and other hospital personnel enter baseline patient demographic and clinical variables, as well as procedural and postoperative variables, into a web-based data collection platform. Because data are collected under the auspices of the SVS Patient Safety Organization, requirements for patient consent were waived. This study was approved by the SVS-VQI Research Advisory Committee. In addition, the SVS-VQI Research Advisory Committee waived the need for patient informed consent because the data were provided in an unlinked, deidentified manner. This study followed the Strengthening the Reporting of Observational Studies in Epidemiology (STROBE) reporting guideline for cross-sectional studies.

**Figure 1.**
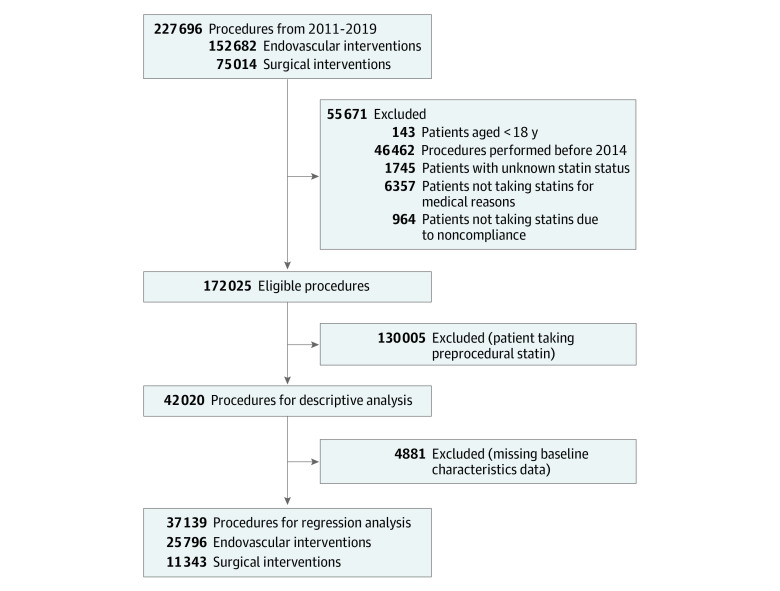
Patient Selection for Final Analyses

All patients at least 18 years of age undergoing LE arterial revascularization from January 1, 2011, to February 7, 2020, were initially considered. A total of 227 696 consecutive procedures in 162 428 patients were performed in over 350 academic and community hospitals representing 45 states. Records in the VQI correspond with individual procedures; a patient with multiple procedures contributes 1 record per event. Due to the high degree of missing data in the early years of collection, we limited our analysis to procedures completed from January 1, 2014, through December 31, 2019. A small number of cases performed in early 2020 were included in 2019 for purposes of analysis. Patients on a statin before their procedure had an expected high rate of statin therapy on discharge (97%). Given this outcome and our interest in identifying factors that influenced the new prescription of statin therapy, patients on baseline statin therapy (n = 130 005) were excluded from logistic regression analyses. Patients not prescribed statin therapy at baseline were also excluded if they were not receiving statins for a medical reason or if they had a history of noncompliance so as to not misrepresent the number of patients in whom physicians could consider initiating stating therapy. After the application of exclusion criteria, 42 020 procedures were included for descriptive analyses, and after removal of missing demographic data, 37 139 procedures corresponding to 30 865 patients remained for multivariate analysis (Figure 1).

### Patient Data

Demographic information on age, sex, race and ethnicity (ie, Black, White, or other race, which included American Indian or Alaska Native, Asian [continent], Indian [subcontinent], and Native Hawaiian or Other Pacific Islander), body mass index (calculated as weight in kilograms divided by height in meters squared), and insurance status (Medicare, Medicaid, commercial, or other) were collected for all procedures. Racial and ethnic categories were reported by the investigator based on available data (ie, patient reported or investigator observed allowed). These data were a part of standard collection practices from the VQI data set; we chose to include this information in our analysis to determine if there was any suggestion of racial or ethnic disparities in care.

Baseline use of antiplatelet therapies, angiotensin-converting enzyme inhibitors (ACEIs), angiotensin receptor blockers (ARBs), and statins were also collected. Clinical variables of interest included smoking status, hypertension, diabetes, coronary heart disease (CHD), heart failure, chronic obstructive pulmonary disease, kidney impairment, and prior peripheral revascularization. Smoking status was categorized as current (use within the last month), prior (use >1 month from intervention), or never. Patients with a documented history of diabetes had their diabetes further classified as diet controlled, requiring oral medications or noninsulin injectables, or requiring insulin. Coronary heart disease was defined as having a history of coronary artery revascularization, myocardial infarction, angina, or a documented stress test result that was positive for ischemia or infarction. Heart failure and chronic obstructive pulmonary disease were defined by a documented history regardless of current functional status. Kidney impairment was defined by an estimated glomerular filtration rate of less than 30 mL/min/1.73 m^2^, derived from the abbreviated Modification of Diet in Renal Disease equation, or the need for dialysis.^15,16^

Procedural-related variables included indication for intervention (ie, asymptomatic, claudication, chronic limb-threatening ischemia, or acute limb ischemia), urgency (ie, elective vs nonelective), type of intervention (ie, endovascular or open surgical repair), and intervention location (ie, suprainguinal or infrainguinal).

### Statistical Analysis

To report differences in baseline characteristics between groups, *t* tests and χ^2^ tests were performed. Multivariable logistic regression was used to determine factors associated with new statin prescription on discharge, with different models built for peripheral and surgical groups. Patients with complete data for all variables (listwise deletion) were included in the main analysis. Approximately 6274 entries (17%) were the result of duplicate procedures. A generalized estimation equation was used to account for these patients with multiple procedures nested within the same hospitals. All demographic variables were included in the final model for analysis. Body mass index was categorized by clinically recognized thresholds. Continuous variable linearity assumptions were tested by fractional polynomial testing. With approximately 10% of missing data, we conducted the same analysis using data with multiple imputations of missing values to address bias and loss of power. A total of 20 data sets were imputed using the fully conditional specification method. Variables included in the imputation model are outcome variables, all independent variables in the final analysis, and surgery year. Statistical significance was defined by a 2-sided *P* value of .05. Analyses were conducted using SAS software, version 9.4 (SAS Institute Inc).

## Results

There were 172 025 procedures corresponding to 125 791 patients (mean [SD] age, 67.7 [11.0] years; 107 800 men [62.7%]; 64 222 women [37.3%]; 26 978 Black [15.7%]; 135 405 White [78.7%]; and 9524 other race [5.5%], which included American Indian or Alaska Native, Asian [continent], Indian [subcontinent], and Native Hawaiian or Other Pacific Islander) included in the descriptive analysis. Baseline characteristics of these patients are outlined in eTable 1 in the Supplement, and a comparison of characteristics between endovascular and surgical intervention showed no significant differences (eTable 2 in the Supplement).

After the application of exclusion criteria, 42 020 procedures corresponding to 34 756 patients were included for multivariate analysis (Table 1). These individuals were older (mean [SD] age, 67.1 [12.6] years), 59.4% men, 16.8% Black, 77.4% White, and 5.6% other race. Approximately 12 790 of 42 020 patients (30%) were newly discharged with a statin after the intervention. Patients not taking a statin postprocedure tended to be older (mean [SD] age, 67.6 [13.0] years vs 65.9 [11.7] years), undergoing elective intervention (80.7% vs 72.1%), and less likely to present with either chronic limb ischemia (52.5% vs 56.7%) or acute limb ischemia (10.4% vs 14.1%). These patients had a lower body mass index (mean [SD], 26.6 [5.6] vs 26.9 [5.6]) and a lower prevalence of cardiovascular comorbidities: smoking (current, 39.6% vs 46.9%), diabetes (diet controlled, 4.7% vs 5.1%), hypertension (78.7% vs 80.7%), and CHD (25.9% vs 30.0%). These patients were also more likely to have had a history of prior peripheral revascularization (43.9% vs 38.4%) and to be receiving antiplatelet therapy (64.1% vs 56.9%).

**Table 1.** Baseline Characteristics of Patients Not Receiving Statin Therapy at Time of Intervention

Variable	Statin postprocedure, No. (%)	
	Yes (n = 12 790)	No (n = 29 230)
Age, mean (SD), y	65.9 (11.7)	67.6 (13.0)
Sex		
Male	8116 (63.5)	16 861 (57.7)
Female	4674 (36.5)	12 367 (42.3)
BMI, mean (SD)	26.9 (5.6)	26.6 (5.6)
Race		
Black	2237 (17.5)	4842 (16.6)
White	9703 (75.9)	22 840 (78.1)
Other^a^	831 (6.5)	1533 (5.3)
Region		
North	4170 (32.6)	8122 (27.8)
East	4376 (34.2)	12 588 (43.1)
South	2591 (20.3)	5212 (17.8)
West	1624 (12.7)	3301 (11.3)
Insurance		
Medicare	6019 (47.1)	15 011 (51.4)
Medicaid	1309 (10.2)	2178 (7.5)
Commercial	4610 (36.0)	10 812 (37.0)
Other	743 (5.8)	1121 (3.8)
Smoking		
Current	5997 (46.9)	11 573 (39.6)
Prior	4337 (33.9)	10 781 (36.9)
Never	2433 (19.0)	6839 (23.4)
Diabetes		
Diet controlled	649 (5.1)	1379 (4.7)
Oral medications	1810 (14.2)	3800 (13.0)
Insulin dependent	3018 (23.6)	5967 (20.4)
No	7307 (57.1)	18 073 (61.8)
Hypertension	10 323 (80.7)	23 016 (78.7)
Coronary heart disease	3835 (30.0)	7583 (25.9)
Heart failure	1841 (14.4)	4105 (14.0)
COPD	3288 (25.7)	7324 (25.1)
Renal impairment	318 (2.5)	735 (2.5)
Prior peripheral revascularization	4916 (38.4)	12 831 (43.9)
Antiplatelet therapy	7283 (56.9)	18 734 (64.1)
ACEI or ARB	4825 (37.7)	11 218 (38.4)
Indication		
Asymptomatic	254 (2.0)	890 (3.0)
Claudication	3454 (27.0)	9891 (33.8)
CLTI	7246 (56.7)	15 337 (52.5)
Acute ischemia	1798 (14.1)	3017 (10.4)
Urgency		
Elective	9220 (72.1)	23 574 (80.7)
Nonelective	3555 (27.8)	5622 (19.2)
Setting^b^		
Inpatient	3128 (24.4)	5145 (17.6)
Outpatient	1776 (13.8)	7604 (26.0)

Abbreviations: ACEI, angiotensin-converting enzyme inhibitor; ARB, angiotensin receptor blocker; BMI, body mass index (calculated as weight in kilograms divided by height in meters squared); CLTI, chronic limb-threatening ischemia; COPD, chronic obstructive pulmonary disease.

^a^ Other race includes American Indian or Alaska Native, Asian (continent), Indian (subcontinent), and Native Hawaiian or Other Pacific Islander.

^b^ Only refers to patients undergoing endovascular intervention.

**Figure 2.**
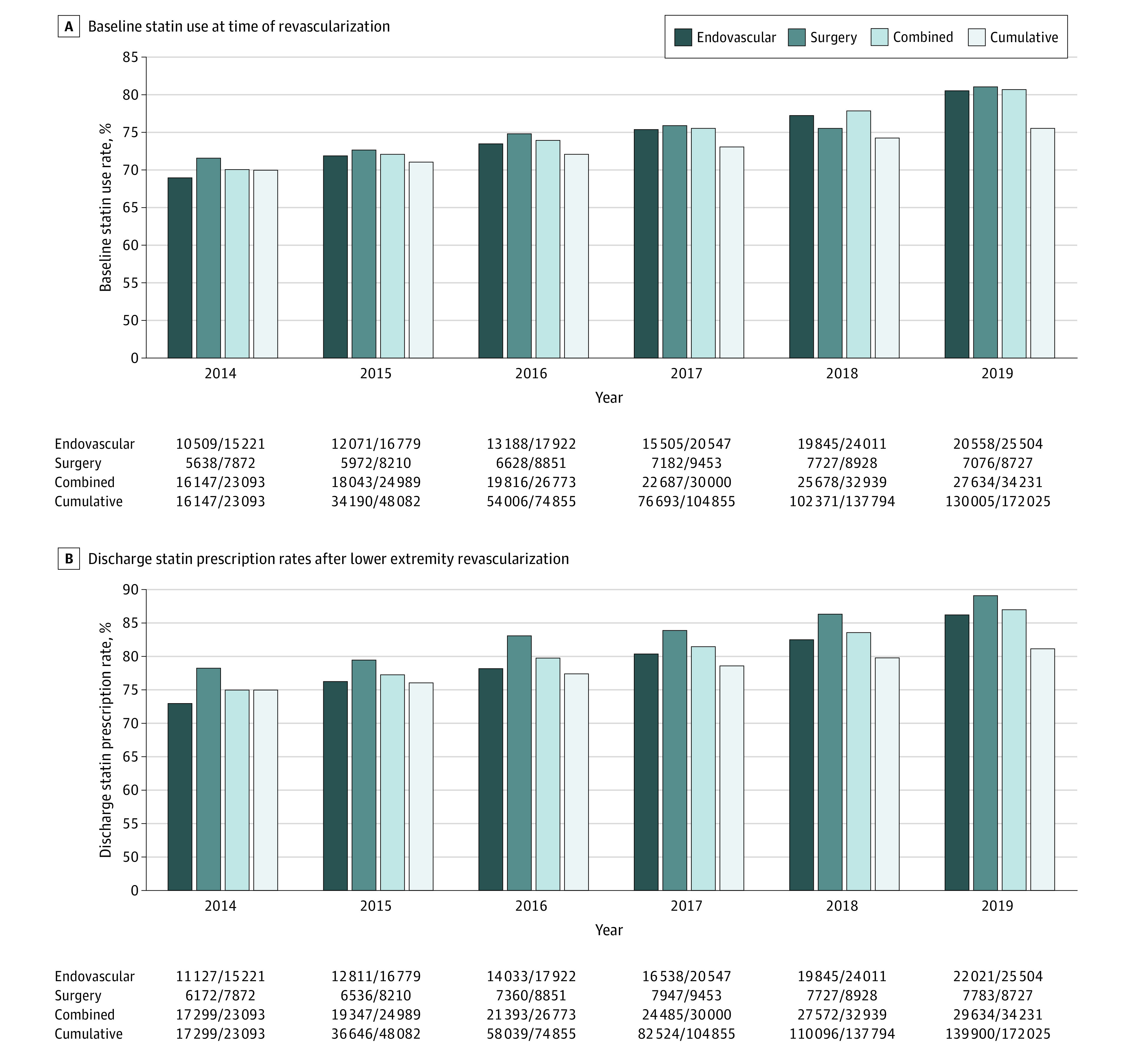
Trends in Statin Use and Prescription Rates Over Time in All Patients Undergoing Lower Extremity Revascularization A, Baseline statin use rates and B, discharge statin prescription rates over time.

Figure 2 illustrates longitudinal trends in statin use for the study population taken at 2 distinct time points: preprocedure (panel A) and at discharge (panel B). Preprocedure statin use increased from 70% to 81% between 2014 and 2019, and percentages were similar when endovascular intervention and LEB were looked at separately. At the time of discharge, 75% of patients (17 299 of 23 093) were prescribed statin therapy in 2014, and this rate increased in a linear fashion to 87% (29 804 of 34 231) in 2019. Discharge statin prescription rates were consistently higher for LEB than for endovascular intervention throughout the study period.

**Figure 3.**
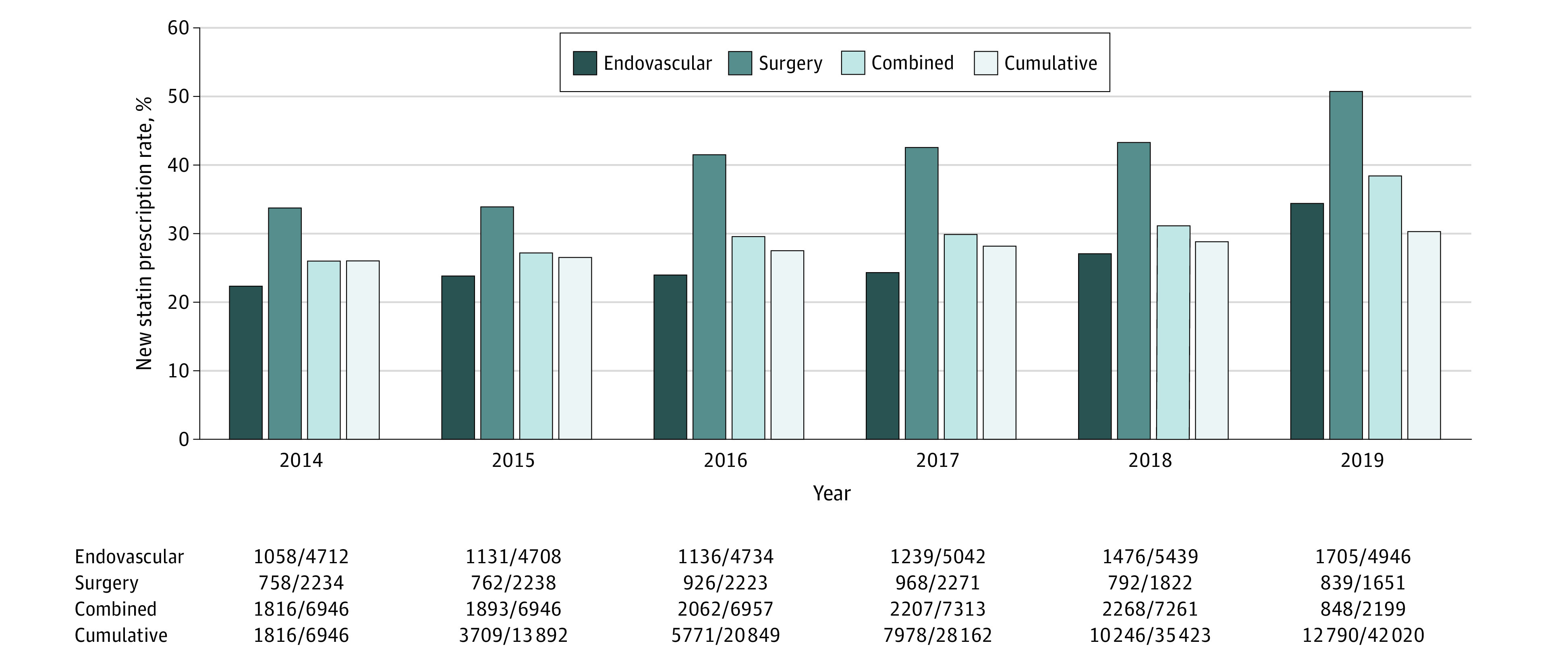
New Statin Prescription Rates After Lower Extremity Revascularization Over Time in Patients Not Receiving Baseline Statin Therapy

Longitudinal trends in new statin prescriptions following LE revascularization are presented in Figure 3. Although progressive annual improvements in new statin prescriptions were noted from 2014 (1816 of 6946 [26.1%]) to 2019 (848 of 2199 [38.6%]), over 60% of patients not receiving baseline therapy were still not prescribed statin therapy after the intervention in 2019. In addition, significant differences were observed based on intervention type: new statin prescription rates rose 12% for patients undergoing endovascular intervention, attaining a peak of 34% in 2019. Patients who underwent LEB had a much higher baseline rate of new statin prescription (34% in 2014) and a greater increase in this rate (17%) by the end of follow-up.

The associations of baseline characteristics with likelihood of new statin prescription after LE endovascular intervention and LEB are reported in Table 2. New statin prescription rates were substantially worse after endovascular intervention (7745 of 29 581 [26%]) than after LEB (5045 of 12 439 [41%]). A lower likelihood of new statin prescription for both the endovascular intervention and LEB groups, respectively, was associated with female sex (odds ratio [OR], 0.90; 95% CI, 0.84-0.96; *P* = .001 and OR, 0.87; 95% CI, 0.80-0.95; *P* < .001), prior peripheral revascularization (OR, 0.76; 95% CI, 0.72-0.81; *P* < .001 and OR, 0.78; 95% CI, 0.72-0.85; *P* < .001), and antiplatelet therapy (OR, 0.75; 95% CI, 0.71-0.80; *P* < .001 and OR, 0.82; 95% CI, 0.76-0.90; *P* < .001). Residing in the northern region (OR, 1.51; 95% CI, 1.40-1.62; *P* < .001 and OR, 1.62; 95% CI, 1.46-1.79; *P* < .001), and cardiovascular comorbidities including diabetes (oral medications vs no diabetes, OR, 1.15; 95% CI, 1.05-1.26; P<.001 and OR 1.35; 95% CI, 1.19, 1.53; P<.001; insulin dependent vs no diabetes, OR, 1.30; 95% CI 1.20 1.42; P<.001; and OR, 1.31; 95% CI 1.16, 1.48; P <.001), smoking (current vs never, OR, 1.32; 95% CI, 1.21-1.45; *P* < .001 and OR, 1.44; 95% CI, 1.27-1.64; *P* < .001), hypertension (OR, 1.19; 95% CI, 1.09-1.29; *P* < .001 and OR, 1.21; 95% CI, 1.09-1.35; *P* < .001), and CHD (OR, 1.26; 95% CI, 1.17-1.35; *P* < .001 and OR, 1.45; 95% CI, 1.31-1.59; *P* < .001), in addition to nonelective indications chronic limb ischemia (OR, 1.20; 95% CI, 1.04-1.12; *P* < .001 and OR, 1.22; 95% CI, 1.11-1.35; *P* < .001) and acute ischemia (OR, 1.23; 95% CI, 1.06-1.38; *P* < .001), were associated with a higher likelihood of statin prescription at discharge for both the endovascular intervention and LEB groups, respectively. In those undergoing endovascular intervention, older age (≥78.1 years, OR, 0.76; 95% CI, 0.68-0.84; *P* < .001) and ACEI or ARB use (OR, 0.92; 95% CI, 0.86-0.98; *P* = .01) were associated with a lower likelihood of being newly discharged with a statin prescription, whereas a body mass index greater than 30 (OR, 1.13; 95% CI, 1.04-1.24; *P* < .001) and acute ischemia (OR, 1.23; 95% CI, 1.06-1.38; P<.001) were associated with an increased likelihood. In those undergoing LEB, the presence of chronic obstructive pulmonary disease (OR, 0.89; 95% CI, 0.81-0.97; *P* = .01) was associated with a lower likelihood of new statin prescription, whereas Black race (OR, 1.18; 95% CI, 1.05-1.32; *P* < .001) was associated with an increased likelihood. For endovascular procedures, the inpatient setting conferred much higher rates of new statin prescription compared to the outpatient setting (37.8% vs 18.9%; *P* < .01). Similar results were observed after including procedures with missing data in multiple imputation analysis (eTable 3 in the Supplement).

**Table 2.** Multivariate Analysis of Factors Associated With the Prescription of New Statin Therapy Following Lower Extremity Intervention

Variable	Endovascular intervention (n = 25 796)			Surgical intervention (n = 11 343)		
	No. (%)	OR (95% CI)	*P* value	No. (%)	OR (95% CI)	*P* value
Age, y^a^						
≤59.0 (≤57.0)	1853/6466 (28.7)	[Reference]	NA	1165/2962 (39.3)	[Reference]	NA
59.1-68.6 (57.1-64.7)	1915/6609 (29.0)	1.09 (1.00-1.19)	.04	1174/2693 (43.6)	1.19 (1.06-1.34)	<.001
68.7-78.0 (64.7-73.0)	1675/6372 (26.3)	1.04 (0.94-1.14)	.47	1275/3017 (42.3)	1.18 (1.04-1.33)	.01
≥78.1 (≥73.1)	1293/6349 (20.4)	0.76 (0.68-0.84)	<.001	988/2671 (37.0)	1.00 (0.87-1.15)	.97
Sex						
Male	4033/14 452 (27.9)	[Reference]	NA	3220/7718 (41.7)	[Reference]	NA
Female	2703/11 344 (23.8)	0.90 (0.84-0.96)	.001	1382/3625 (38.1)	0.87 (0.80-0.95)	<.001
Body mass index (calculated as weight in kilograms divided by height in meters squared)						
≤25	267810 832 (24.7)	[Reference]	NA	1950/4897 (39.8)	[Reference]	NA
25.1-29.9	2133/8302 (25.7)	1.02 (0.95-1.04)	.66	1524/3738 (40.8)	1.04 (0.94-1.14)	.46
≥30	1925/6662 (28.9)	1.13 (1.04-1.24)	<.001	1128/2708 (41.7)	1.05 (0.95-1.17)	.33
Race						
Black	1062/3879 (27.4)	1.07 (0.98-1.17)	.11	784/1691 (44.2)	1.18 (1.05-1.32)	<.001
White	5203/20 423 (25.5)	[Reference]	NA	3633/9178 (39.6)	[Reference]	NA
Region						
East	2440/10 866 (22.5)	[Reference]	NA	1441/4103 (35.1)	[Reference]	NA
North	2377/8066 (29.5)	1.51 (1.40-1.62)	<.001	1427/3052 (46.8)	1.62 (1.46-1.79)	<.001
South	964/3592 (26.8)	1.22 (1.11-1.34)	<.001	1254/3080 (40.7)	1.21 (1.09-1.34)	<.001
West	955/3272 (29.2)	1.46 (1.32-1.60)	<.001	480/1108 (43.3)	1.50 (1.29-1.73)	<.001
Insurance						
Medicare	3268/13 174 (24.8)	[Reference]	NA	1970/4881 (40.4)	[Reference]	NA
Medicaid	655/1968 (33.3)	1.19 (1.06-1.35)	<.001	534/1187 (45.0)	1.13 (0.97-1.31)	.12
Commercial	2408/9586 (25.1)	0.96 (0.89-1.03)	.23	1834/4641 (39.5)	0.96 (0.88-1.06)	.47
Other	405/1068 (37.9)	1.48 (1.28-1.71)	<.001	264/634 (41.6)	0.98 (0.81-1.18)	.84
Smoking						
Never	1407/6021 (23.4)	[Reference]	NA	582/1661 (35.0)	[Reference]	NA
Current	2997/10 312 (29.1)	1.32 (1.21-1.45)	<.001	2510/5774 (43.5)	1.44 (1.27-1.64)	<.001
Prior	2332/9463 (24.6)	1.16 (1.06-1.26)	<.001	1510/3908 (38.6)	1.21 (1.07-1.38)	<.001
Diabetes						
No	3734/15 613 (23.9)	[Reference]	NA	3005/7853 (38.3)	[Reference]	NA
Diet controlled	322/1099 (29.3)	1.22 (1.05-1.41)	.01	199/456 (43.6)	1.17 (0.96-1.44)	.12
Oral medications	1014/3736 (27.1)	1.15 (1.05-1.26)	<.001	618/1343 (46.0)	1.35 (1.19-1.53)	<.001
Insulin dependent	1666/5348 (31.1)	1.30 (1.20-1.42)	<.001	780/1691 (46.1)	1.31 (1.16-1.48)	<.001
Hypertension	5413/20 378 (26.6)	1.19 (1.09-1.29)	<.001	3662/8725 (42.0)	1.21 (1.09-1.35)	<.001
Coronary heart disease	1950/6835 (28.5)	1.26 (1.17-1.35)	<.001	1330/2846 (46.7)	1.45 (1.31-1.59)	<.001
Heart failure	972/3441 (28.2)	1.07 (0.98-1.17)	.15	467/1107 (42.2)	0.94 (0.81-1.08)	.35
COPD	1709/6342 (26.9)	1.04 (0.97-1.12)	.28	1209/3053 (39.6)	0.89 (0.81-0.97)	.001
Renal impairment	211/766 (27.5)	0.95 (0.80-1.13)	.56	92/237 (38.8)	0.88 (0.67-1.15)	.34
Prior revascularization	2387/10 521 (22.7)	0.76 (0.72-0.81)	<.001	1977/5263 (37.6)	0.78 (0.72-0.85)	<.001
Antiplatelet therapy	3851/16 376 (23.5)	0.75 (0.71-0.80)	<.001	2599/6771 (38.4)	0.82 (0.76-0.90)	<.001
ACEI or ARB	2628/10 356 (25.4)	0.92 (0.86-0.98)	.01	1747/4167 (41.9)	1.02 (0.93-1.11)	.69
Indication						
Claudication	2147/9627 (22.3)	[Reference]	NA	1113/2988 (37.2)	[Reference]	NA
Asymptomatic	123/636 (19.3)	0.84 (0.68-1.11)	.09	111/422 (26.3)	0.63 (0.50-0.80)	<.001
CLTI	3628/13 107 (27.7)	1.20 (1.04-1.12)	<.001	2572/6014 (42.8)	1.22 (1.11-1.35)	<.001
Acute ischemia	838/2426 (34.5)	1.23 (1.06-1.38)	<.001	806/1919 (42.0)	1.13 (0.99-1.30)	.08
Urgency						
Elective	4833/20 628 (23.4)	[Reference]	NA	3368/8576 (39.3)	[Reference]	NA
Nonelective	1903/5168 (36.8)	1.60 (1.48-1.73)	<.001	1234/2767 (44.6)	1.18 (1.06-1.30)	<.001

Abbreviations: ACEI, angiotensin-converting enzyme inhibitor; ARB, angiotensin receptor blocker; CLTI, chronic limb-threatening ischemia; COPD, chronic obstructive pulmonary disease; NA, not applicable; OR, odds ratio.

^a^ Age reported by quartiles, with surgical intervention quartiles shown in parentheses.

## Discussion

Despite substantial improvements in statin use for patients undergoing LE revascularization over a relatively short period of time, significant treatment gaps remain. Participation in quality improvement registries, such as the VQI, has been shown to improve medical management in accordance with guidelines; as such, our study may actually have overestimated the true percentage of patients being discharged with statin therapy after their procedure.^17^ Despite this improvement, results of this cross-sectional study suggest that less than one-third of patients not already on a statin at the time of revascularization were discharged with one. This disparity was worse for those undergoing endovascular intervention compared with LEB (26% vs 41%). We identified a variety of demographic and clinical factors associated with the likelihood of a new statin prescription at discharge.

Although statin discharge rates after LE revascularization approached 90% by 2019, new statin prescription rates remained low at the study’s end. Only one-third of eligible individuals were newly discharged with a statin after endovascular intervention compared with one-half of individuals being discharged with a statin after LEB. This outcome is an important finding considering the growing preference for endovascular intervention over LEB as the default revascularization approach.^18,19^ Many factors may be underlying this discrepancy. All surgical interventions required inpatient hospitalization, whereas endovascular interventions occurred in both the inpatient and outpatient settings. Rates of new statin prescriptions were significantly higher for inpatient endovascular procedures than for outpatient procedures. Presumably, in the outpatient setting, the operator would be solely responsible for prescribing guideline-appropriate medications on discharge, whereas this responsibility may be shared among the various health care professionals (hospitalists, vascular medicine specialists, or inpatient pharmacists) in the inpatient setting, thereby resulting in a higher likelihood of statin prescription. In addition, a hospitalization may allow more time for a patient-physician conversation regarding statin initiation and a higher degree of ownership by the inpatient clinicians with regard to this important decision. Unfortunately, owing to 40% missing data, we were unable to include treatment setting in the multivariate analysis to formally determine whether this factor was associated with new statin prescription. In addition, clinical practice guidelines give a class IIA recommendation (ie, indicates that the recommendation should be considered as current evidence and expert opinion suggest usefulness) for perioperative statin initiation in patients specifically undergoing vascular surgery for cardiovascular and limb benefit, and this specification may also account for some of the difference noted.^3,4,5,20,21,22,23^

New statin prescription in patients with PAD was more likely in those with traditional cardiovascular risk factors and established polyvascular disease, most notably CHD. This finding is in line with previous studies from the Veterans Affairs registry and National Health and Nutrition Examination Survey.^9,24,25^ Importantly, prior data have demonstrated that patients with polyvascular disease do not experience a significantly improved statin benefit for limb outcomes compared with patients with isolated PAD.^12^ Our study identified an important area for improvement, as a lack of cardiovascular risk factors or the presence of established CHD does not make PAD less concerning, particularly in a patient population that is presenting for revascularization due to significant symptoms. Patients undergoing intervention for nonelective indications had higher rates of statin prescription in both the surgical and endovascular cohorts. Similar findings were noted in earlier analysis of the VQI, which found that rates of optimal goal-directed medical therapy were significantly lower for those undergoing peripheral revascularization for intermittent claudication compared with chronic limb ischemia.^26^ Fear of adverse events from statin therapy given that this would mimic symptoms of the disease process as well as a possible belief from clinicians that claudication alone doesn’t warrant aggressive treatment despite significant symptoms requiring revascularization may be underlying factors.

New statin prescription rates were lower in women, consistent with prior studies demonstrating underrecognition and undertreatment of atherosclerotic cardiovascular disease in this population.^27^ Women often present with atypical symptoms, which may result in delayed referrals and intervention, and higher rates of more complex disease.^28,29^ Reasons for underprescription in this population may reflect dismissal of symptoms given atypical presentation, as well as implicit bias from clinicians. Prior studies have shown that women are less likely to survive cardiac disease when treated by male clinicians; however, when treated by female clinicians, there is no significant difference in outcomes between the sexes, presumably due to better vigilance for atypical symptoms, higher awareness of disease consequence in women, and resultant improvement in medical therapy.^30^ Women have also been underrepresented in clinical trials, and outcomes may be less understood, but in available data from meta-analyses, both sexes seem to benefit from statin therapy with regard to atherosclerotic vascular disease.^31^

Patients undergoing endovascular interventions were older than those undergoing surgical repair in our study. An age older than 78 years was associated with reduced likelihood of new statin prescription in those undergoing endovascular intervention. This finding may be due to concerns regarding polypharmacy and a perceived increased risk of side effects, such as rhabdomyolysis due to poor clearance and comorbid conditions, which results in a lower benefit-to-risk ratio.^32,33^ Although an individualized approach should be used in patients with advanced age, there is growing evidence that suggests a similar protective benefit regardless of age.^34^

Despite excluding individuals who were not taking a statin before their procedure due to medical reasons or noncompliance, patients may have been presumed to not be taking statins due to unwillingness, intolerance, or an inability to afford medications, and operators may have deferred these discussions to their continuity care professionals. This finding is supported by the fact that patients with prior interventions and not receiving statin therapy were less likely to be discharged with new statin therapy compared with those presenting for initial intervention. Similar findings have been reported in patients with heart failure, in which initiation of therapy is often delayed to the outpatient setting, and there is significant underprescription even after outpatient follow-up.^35,36^ Further efforts to better identify and understand these barriers are important as this was not possible to adequately assess in the VQI.

### Limitations

This study had several limitations. Due to inadequate follow-up data, we were unable to comment on differences in adverse cardiac or limb outcomes according to new statin discharge prescription status. The 3 major groups performing endovascular intervention were vascular surgeons, interventional radiologists, and interventional cardiologists; however, clinician-specific prescription patterns could not be performed as individual operator data for specialty type or volume were not made available.

Although we attempted to exclude all individuals not receiving statin therapy due to medical reasons or noncompliance, it is possible that some may not have been thoroughly screened during the data capture process, which was performed by individual clinicians, and were inappropriately included. Therefore, our findings may have been slightly exaggerated.

The VQI does not capture the intensity of statin therapy, which may have important clinical implications. High-intensity statin therapy is recommended for those with clinical PAD unless the therapy cannot be tolerated, and it has a clear cardiovascular benefit in this population.^37^ Increasing statin intensity has been shown to further reduce amputation risk.^10^ Although our study highlights suboptimal use of statin therapy, when considering the intensity of statin therapy, appropriate rates are likely to be much lower.

In addition, the ratio of endovascular to surgical interventions in the VQI are lower than what has been reported in Medicare populations.^38,39^ If the true rate of endovascular interventions is greater than reported in the VQI, our findings are even more concerning as this would suggest that overall rates of new statin therapy are lower than those reported here.

## Conclusions

Despite improvements in statin use for patients following LE revascularization, these cross-sectional study results suggest that substantial underprescription remains, particularly in those not already taking a statin preprocedurally. New statin prescriptions were particularly low in those undergoing endovascular intervention, which has important clinical implications given the current trend toward a default endovascular approach. Further studies to better understand barriers to physician prescription of statins in this population is warranted, as well as investigations to understand resultant clinical implications. Clinician- and system-based interventions, such as implementing a “Get With the Guidelines” check-and-review system that addresses this deficiency, are needed to improve overall compliance.^40,41^

## Notes

Supplement. **eTable 1.** Baseline Characteristics of Patients Undergoing Lower Extremity Revascularization **eTable 2.** Baseline Characteristics of Patients Presenting for Revascularization Based on Intervention Type **eTable 3.** Multiple Imputation Analysis of Factors Associated With the Prescription of New Statin Therapy Following Lower Extremity Intervention
