# Supplementary material for: Prescribing of Statins After Lower Extremity Revascularization Procedures in the US
Source: JAMA Netw Open. 2021 Dec 3;4(12):e2136014. doi: 10.1001/jamanetworkopen.2021.36014 (PMC8642785; doi:10.1001/jamanetworkopen.2021.36014)
Supplement: Supplement. — eTable 1. Baseline Characteristics of Patients Undergoing Lower Extremity Revascularization eTable 2. Baseline Characteristics of Patients Presenting for Revascularization Based on Intervention Type eTable 3. Multiple Imputation Analysis of Factors Associated With the Prescription of New Statin Therapy Following Lower Extremity Intervention [file jamanetwopen-e2136014-s001.pdf]

# Supplementary Online Content

Singh N, Ding L, Devera J, Magee GA, Garg PK. Prescribing of statins after lower extremity revascularization procedures in the US. *JAMA Netw Open*. 2021;4(12):e2136014. doi:10.1001/jamanetworkopen.2021.36014

**eTable 1.** Baseline Characteristics of Patients Undergoing Lower Extremity Revascularization

**eTable 2.** Baseline Characteristics of Patients Presenting for Revascularization Based on Intervention Type

**eTable 3.** Multiple Imputation Analysis of Factors Associated With the Prescription of New Statin Therapy Following Lower Extremity Intervention

This supplementary material has been provided by the authors to give readers additional information about their work.

© 2021 Singh N et al. *JAMA Network Open.*

**eTable 1.** Baseline Characteristics of Patients Undergoing Lower-Extremity Revascularization

| Variable | Statin post-procedure (n = 139,900) | No statin post-procedure (n = 32,125) | P-Value |
|---|---|---|---|
| **Age (years)** | 67.6 ± 10.6 | 67.7 ± 12.9 | 0.28 |
| **Sex** | | | < 0.01 |
| Male | 89,169 (63.7) | 18,631 (58.0) | |
| Female | 50,730 (36.3) | 13,492 (42.0) | |
| **BMI (kg/m$^2$)** | 27.9 ± 5.5 | 26.7 ± 5.6 | < 0.01 |
| **Race** | | | < 0.01 |
| White | 110,348 (78.9) | 25,057 (78.0) | |
| Black | 21,629 (15.5) | 5,348 (16.7) | |
| Other | 7,822 (5.6) | 1,702 (5.3) | |
| **Region** | | | < 0.01 |
| North | 45,893 (32.8) | 8,850 (27.6) | |
| East | 53,841 (38.5) | 13,903 (43.3) | |
| South | 26,736 (19.1) | 5,823 (18.1) | |
| West | 13,307 (9.5) | 3,539 (11.0) | |
| **Insurance** | | | 0.28 |
| Medicare | 71,273 (51.0) | 16,589 (51.6) | |
| Medicaid | 10,518 (7.5) | 2,368 (7.4) | |
| Commercial | 52,766 (37.0) | 11,807 (36.8) | |
| Other | 5,273 (3.8) | 1,241 (3.9) | |
| **Smoking** | | | < 0.01 |
| Current | 50,084 (35.8) | 12,602 (39.2) | |
| Prior | 65,176 (46.6) | 12,044 (37.5) | |
| Never | 24,509 (17.5) | 7,440 (23.2) | |
| **Diabetes** | | | < 0.01 |
| Diet-controlled | 6,144 (4.4) | 1,498 (4.7) | |
| Oral Medications | 25,265 (18.1) | 4,257 (13.3) | |
| Insulin-Dependent | 42,802 (30.6) | 6,941 (21.6) | |
| No | 65,636 (46.9) | 19,418 (60.5) | |
| **Hypertension** | 125,685 (89.8) | 25,621 (79.8) | < 0.01 |
| **Coronary Heart Disease** | 68,981 (49.3) | 8,901 (27.7) | < 0.01 |
| **Heart Failure** | 27,231 (19.5) | 4,700 (14.6) | < 0.01 |
| **COPD** | 39,612 (28.3) | 8,154 (25.4) | < 0.01 |
| **Renal Impairment** | 3,805 (2.7) | 850 (2.7) | 0.69 |
| **Prior Peripheral Revascularization** | 74,798 (53.5) | 12,293 (44.5) | < 0.01 |

© 2021 Singh N et al. *JAMA Network Open.*

| | | | |
|---|---|---|---|
| **Antiplatelet Therapy** | 118,138 (84.4) | 21,059 (65.6) | < 0.01 |
| **ACE-I or ARB** | 77,041 (55.1) | 12,632 (39.3) | < 0.01 |
| **Indication** | | | < 0.01 |
| Asymptomatic | 3,958 (2.8) | 966 (3.0) | |
| Claudication | 54,755 (39.1) | 10,785 (33.6) | |
| CLTI | 70,365 (50.3) | 16,881 (52.6) | |
| Acute Ischemia | 10,536 (7.5) | 3,398 (10.6) | |
| **Urgency** | | | < 0.01 |
| Elective | 116,485 (83.3) | 25,797 (80.3) | |
| Non-Elective | 23,281 (16.6) | 6,289 (19.6) | |

BMI = body mass index, COPD = chronic obstructive pulmonary disease, ACE-I = angiotensin converting enzyme inhibitor, ARB = angiotensin receptor blocker, CLTI = chronic limb-threatening ischemia

© 2021 Singh N et al. *JAMA Network Open.*

**eTable 2.** Baseline Characteristics of Patients Presenting for Revascularization Based on Intervention Type

| Variable | Surgical Intervention (n = 12,439) | | Endovascular Intervention (n = 29,581) | |
|---|---|---|---|---|
| | Post-Procedure Statin (n=5,045) | No Post-Procedure Statin (n=7,394) | Post-Procedure Statin (n=7,745) | No Post-Procedure Statin (n=21,836) |
| **Age (years)** | 64.5 ± 11.0 | 64.7 ± 12.9 | 66.8 ± 12.0 | 68.7 ± 12.9 |
| **Sex** | | | | |
| Male | 3,501 (69.4) | 4,892 (66.2) | 4,615 (59.6) | 11,969 (54.8) |
| Female | 1,544 (30.6) | 2,500 (33.8) | 3,130 (40.4) | 9,867 (45.2) |
| **BMI (kg/m$^2$)** | 26.6 ± 5.3 | 26.4 ± 5.4 | 27.2 ± 5.8 | 26.7 ± 5.6 |
| **Race** | | | | |
| White | 3,917 (77.6) | 5,958 (80.6) | 5,786 (74.7) | 16,882 (77.3) |
| Black | 861 (17.1) | 1,139 (15.4) | 1,376 (17.8) | 3,703 (17.0) |
| Other | 253 (5.0) | 289 (3.9) | 578 (7.5) | 1,244 (5.7) |
| **Region** | | | | |
| North | 1,515 (30.0) | 1,754 (23.7) | 2,655 (34.3) | 6,368 (29.2) |
| East | 1,576 (31.2) | 2,885 (39.0) | 2,800 (36.2) | 9,703 (44.4) |
| South | 1,403 (27.8) | 2,073 (28.0) | 1,188 (15.3) | 3,139 (14.4) |
| West | 522 (10.4) | 675 (9.1) | 1,102 (14.2) | 2,626 (12.0) |
| **Insurance** | | | | |
| Medicare | 2,185 (43.3) | 3,297 (44.6) | 3,834 (49.5) | 11,714 (53.7) |
| Medicaid | 580 (11.5) | 683 (9.2) | 729 (9.4) | 1,495 (6.9) |
| Commercial | 1,965 (39.0) | 2,995 (40.5) | 2,645 (34.2) | 7,817 (35.8) |
| Other | 311 (6.2) | 298 (5.4) | 432 (5.6) | 723 (3.3) |
| **Smoking** | | | | |
| Current | 2,706 (53.6) | 3,543 (47.9) | 3,291 (42.5) | 8,030 (36.8) |
| Prior | 1,656 (32.8) | 2,608 (35.3) | 2,681 (34.6) | 8,173 (37.4) |
| Never | 680 (13.5) | 1,242 (16.8) | 1,753 (22.6) | 5,597 (25.6) |
| **Diabetes** | | | | |
| Diet-controlled | 235 (4.7) | 303 (4.1) | 414 (5.4) | 1,076 (4.9) |
| Oral Medications | 675 (13.4) | 787 (10.6) | 1,135 (14.7) | 3,013 (13.8) |
| Insulin-Dependent | 912 (18.1) | 1,113 (15.1) | 2,106 (27.2) | 4,854 (22.2) |
| No | 3,221 (63.9) | 5,190 (70.2) | 4,086 (52.8) | 12,883 (59.0) |
| **Hypertension** | 4,029 (79.9) | 5,615 (75.9) | 6,294 (81.3) | 17,401 (79.7) |
| **Coronary Heart Disease** | 1,511 (30.0) | 1,747 (23.6) | 2,324 (30.0) | 5,836 (26.7) |
| **Heart Failure** | 570 (11.3) | 802 (10.9) | 1,271 (16.4) | 3,303 (15.1) |
| **COPD** | 1,324 (26.2) | 2,034 (27.5) | 1,964 (25.4) | 5,290 (24.2) |

© 2021 Singh N et al. *JAMA Network Open.*

| | | | | |
|---|---|---|---|---|
| **Renal Impairment** | 94 (1.9) | 155 (2.1) | 224 (2.9) | 580 (2.7) |
| **Prior Revascularization** | 2,175 (43.1) | 3,587 (48.5) | 2,741 (35.4) | 9,244 (42.3) |
| **Antiplatelet Therapy** | 2,841 (56.3) | 4,537 (61.4) | 4,442 (57.4) | 14,197 (65.0) |
| **ACE-I or ARB** | 1,870 (37.1) | 2,610 (35.3) | 2.955 (38.2) | 8,608 (39.4) |
| **Indication** | | | | |
| Asymptomatic | 117 (2.3) | 330 (4.5) | 137 (1.8) | 560 (2.6) |
| Claudication | 1,160 (23.0) | 1,963 (26.6) | 2,294 (29.6) | 7,298 (36.3) |
| CLTI | 2,866 (56.8) | 3,854 (52.1) | 4,380 (56.6) | 11,483 (52.6) |
| Acute Ischemia | 883 (17.5) | 1,228 (16.6) | 915 (11.8) | 1,799 (8.2) |
| **Urgency** | | | | |
| Elective | 3,668 (72.7) | 5,665 (76.6) | 5,552 (71.7) | 17,909 (82.0) |
| Non-Elective | 1,368 (27.1) | 1,718 (23.2) | 2,187 (28.2) | 3,904 (17.9) |

BMI = body mass index, COPD = chronic obstructive pulmonary disease, ACE-I = angiotensin converting enzyme inhibitor, ARB = angiotensin receptor blocker, CLTI = chronic limb-threatening ischemia

© 2021 Singh N et al. *JAMA Network Open.*

**eTable 3.** Multiple Imputation Analysis of Factors Associated With the Prescription of New Statin Therapy Following Lower Extremity Intervention

| Variable | Endovascular (n = 29.581) | | Surgical (n = 12,439) | |
|---|---|---|---|---|
| | **OR (95% CI)** | **p-value** | **OR (95% CI)** | **p-value** |
| **Age (years)*** | | | | |
| ≤ 59.0 (≤ 57.0) | Ref | ----- | Ref | ----- |
| 59.1 - 68.6 (57.1-64.7) | 1.12 (1.03 – 1.21) | < 0.01 | 1.18 (1.06 – 1.32) | < 0.01 |
| 68.7 - 78.0 (64.7-73.0) | 1.06 (0.97 – 1.16) | 0.20 | 1.25 (1.11 – 1.40) | < 0.01 |
| ≥ 78.1 (≥ 73.1) | 0.81 (0.73 – 0.89) | < 0.01 | 1.06 (0.93 – 1.20) | 0.37 |
| **Sex** | | | | |
| Male | Ref | ----- | Ref | ----- |
| Female | 0.9 (0.85 – 0.96) | < 0.01 | 0.87 (0.80 – 0.95) | < 0.01 |
| **BMI (kg/m$^2$)** | | | | |
| <=25 | Ref | ----- | Ref | ----- |
| 25.1-29.9 | 1.02 (0.95 – 1.09) | 0.53 | 1.06 (0.97 – 1.16) | 0.21 |
| >=30 | 1.12 (1.04 – 1.21) | < 0.01 | 1.08 (0.98 – 1.20) | 0.13 |
| **Race** | | | | |
| White | Ref | ----- | Ref | ----- |
| Black | 1.05 (0.97 – 1.14) | 0.21 | 1.12 (1.01 – 1.25) | 0.04 |
| **Region** | | | | |
| East | Ref | ----- | Ref | ----- |
| North | 1.50 (1.40 – 1.61) | < 0.01 | 1.57 (1.43 – 1.74) | < 0.01 |
| South | 1.26 (1.16 – 1.38) | < 0.01 | 1.18 (1.07 – 1.30) | < 0.01 |
| West | 1.47 (1.34 – 1.60) | < 0.01 | 1.49 (1.29 – 1.71) | < 0.01 |
| **Insurance** | | | | |
| Medicare | Ref | ----- | Ref | ----- |
| Medicaid | 1.23 (1.10 – 1.37) | < 0.01 | 1.25 (1.08 – 1.44) | < 0.01 |
| Commercial | 1.01 (0.94 – 1.07) | 0.88 | 1.02 (0.93 – 1.11) | 0.72 |
| Other | 1.55 (1.35 – 1.78) | < 0.01 | 1.13 (0.95 – 1.35) | 0.17 |
| **Smoking** | | | | |
| Never | Ref | ----- | Ref | ----- |
| Current | 1.31 (1.21 – 1.43) | < 0.01 | 1.44 (1.27 – 1.62) | < 0.01 |
| Prior | 1.13 (1.05 – 1.22) | < 0.01 | 1.22 (1.08 – 1.38) | < 0.01 |
| **Diabetes** | | | | |
| No | Ref | ----- | Ref | ----- |
| Diet-controlled | 1.14 (1.00 – 1.29 | 0.05 | 1.19 (0.99 – 1.44) | 0.07 |
| Oral Medications | 1.16 (1.07 - 1.27) | < 0.01 | 1.36 (1.21 – 1.54) | < 0.01 |
| Insulin-Dependent | 1.25 (1.16 – 1.25) | < 0.01 | 1.28 (1.14 – 1.43) | < 0.01 |

© 2021 Singh N et al. *JAMA Network Open.*

| | | | | |
|---|---|---|---|---|
| **Hypertension** | 1.16 (1.07 – 1.25) | < 0.01 | 1.19 (1.08 – 1.31) | < 0.01 |
| **Coronary Heart Disease** | 1.25 (1.17 – 1.34) | < 0.01 | 1.44 (1.31 – 1.57) | < 0.01 |
| **Heart Failure** | 1.01 (0.93 – 1.10) | 0.73 | 0.91 (0.80 – 1.04) | 0.16 |
| **COPD** | 1.04 (0.98 – 1.12) | 0.22 | 0.88 (0.80 – 0.96) | 0.01 |
| **Renal Impairment** | 0.93 (0.79 – 1.11) | 0.43 | 0.81 (0.62 – 1.07) | 0.14 |
| **Prior Revascularization** | 0.77 (0.73 – 0.82) | < 0.01 | 0.80 (0.74 – 0.86) | < 0.01 |
| **Antiplatelet Therapy** | 0.78 (0.74 – 0.83) | < 0.01 | 0.83 (0.77 – 0.90) | < 0.01 |
| **ACE-I or ARB** | 0.95 (0.89 – 1.00) | 0.07 | 1.02 (0.93 – 1.11) | 0.68 |
| **Indication** | | | | |
| Claudication | Ref | ----- | Ref | ----- |
| Asymptomatic | 0.85 (0.70 – 1.03) | 0.09 | 0.62 (0.49 – 0.78) | < 0.01 |
| CLTI | 1.20 (1.12 – 1.28) | < 0.01 | 1.23 (1.12 – 1.35) | < 0.01 |
| Acute Ischemia | 1.36 (1.22 – 1.52) | < 0.01 | 1.13 (1.00 – 1.29) | 0.06 |
| **Urgency** | | | | |
| Elective | Ref | ----- | Ref | ----- |
| Non-Elective | 1.55 (1.45 – 1.67) | < 0.01 | 1.16 (1.06 – 1.28) | < 0.01 |

*****Age reported by quartiles, with surgical intervention quartiles shown in parenthesis

BMI = body mass index, COPD = chronic obstructive pulmonary disease, ACE-I = angiotensin converting enzyme inhibitor, ARB = angiotensin receptor blocker, CLTI = chronic limb-threatening ischemia

© 2021 Singh N et al. *JAMA Network Open.*
